# Supplementary material for: Action boosts episodic memory encoding in humans via engagement of a noradrenergic system
Source: Nat Commun. 2019 Aug 6;10:3534. doi: 10.1038/s41467-019-11358-8 (PMC6684634; doi:10.1038/s41467-019-11358-8)
Supplement: Supplementary file 1 — Supplementary Information [file 41467_2019_11358_MOESM1_ESM.pdf]

Action boosts episodic memory encoding in humans via engagement of a noradrenergic system. (Yebra et al., 2019)

Supplementary Information

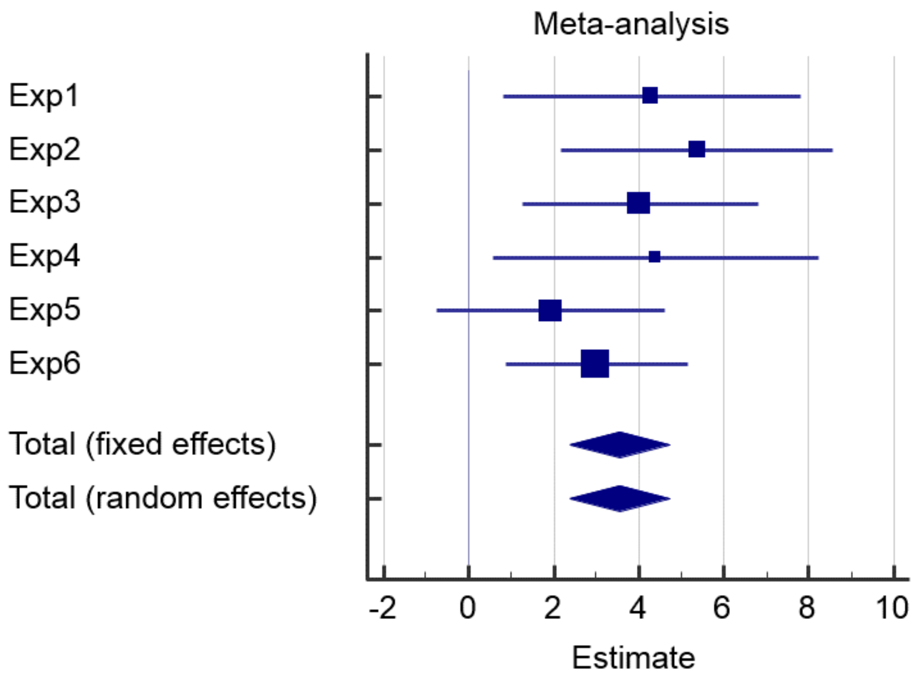

Supplementary Figure 1. Results of meta-analysis across experiments. The differences in mean memory accuracy between stimuli paired with Go *vs.* NoGo responses for Exp 1 to 6 is plotted with 95% confidence intervals (total random effect estimate of 3.57; Wilcoxon signed rank test z = 5.99; $P < 0.001$). Pooled effects (random and fixed) are represented by a diamond, the location of the diamond representing the estimated effect size and the width representing the precision of the estimate. Note that random and fixed models agree when there is no heterogeneity, *i.e.*, no variation in outcomes between experiments (Test for heterogeneity $I^2 = 0.00\%$; Cohran's Q = 3.34, $P = 0.65$).

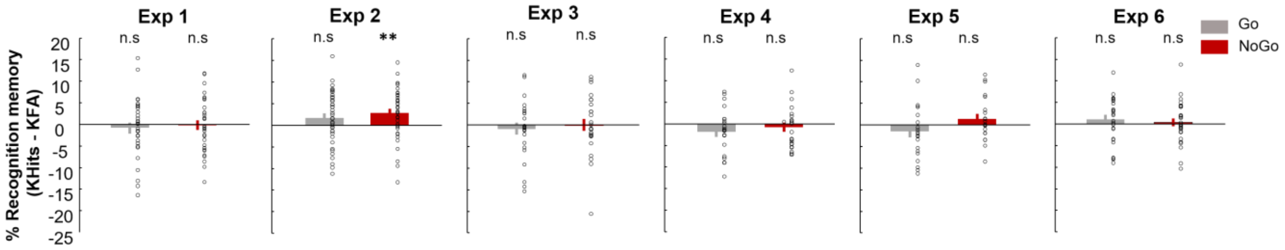

Supplementary Figure 2. (See also Supplementary Table 2) Related to Figure 2. Familiarity recognition behavioral results. Recognition memory for the K responses corrected by false alarms (proportion of know (K) responses on new items). All error bars are the SEM.

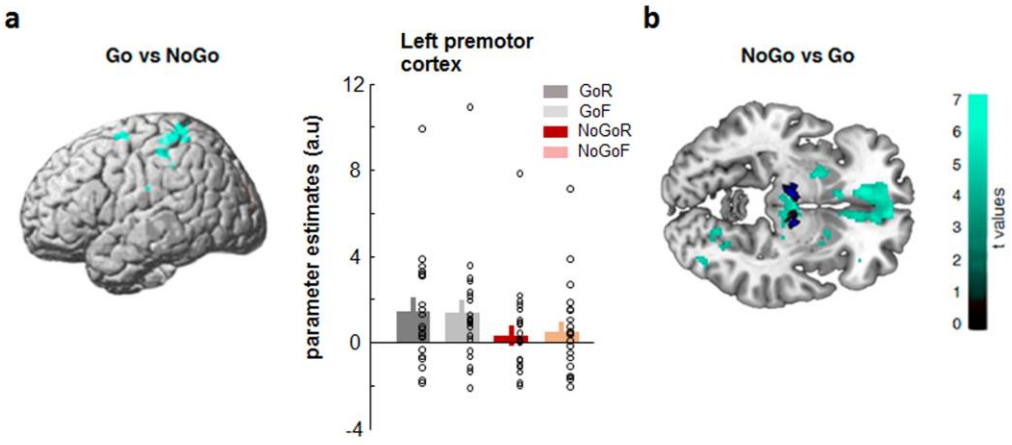

Supplementary Figure 3. (See also Supplementary Table 5, 6). Related to Figure 3. fMRI responses during action (Go) and response inhibition (NoGo).(a) Regions showing a significant Go *vs.* NoGo main effect are rendered onto the left hemisphere of a canonical T1 image (threshold *P* < 0.001 uncorrrected). Parameter estimates for activation in left premotor cortex (*x*, *y*, *z* coordinates -28, 0, 66; *Z* = 3.91; *P* < 0.001 unc.) are plotted to the right. Error bars pertain to s.e.m. (b) Significant NoGo *vs.* Go main effect in an extended neuroanatomical network, including subthalamic nucleus (STN) (6, -18, 2; Z = 5.04, *P* < 0.001 FWE-corrected) The STN has been localized using a probabilistic atlas (dark blue) (Keuken et al., 2014).

Supplementary Table 1. Related to Figure 2. Statistics for reaction times (RT) of Go stimuli as a function of subsequent memory for each Exp. For Exp 7 A and B, the comparison of all subjects collapsed over stimulus emotion (neutral and emotional) is provided first, followed by test for an effect of emotion and the interaction between emotion and encoding success. The latter comparisons are provided first for all subjects, and then grouped according to whether Go-induced memory enhancement was observed for neutral stimuli or not.

**Experiment**		***t*-test RTs GoR *vs*. RTs GoF**
**Exp 1**		t_{30} = -0.73; P = 0.472
**Exp 2**		t_{37} = -1.13; P = 0.267
**Exp 3**		t_{25} = 0.79; P = 0.436
**Exp 4**		t_{21} = 0.53; P = 0.601
**Exp 5**		t_{20} = -0.28; P = 0.781
**Exp 6**		t_{27} = -0.78; P = 0.440
**Exp 7 A**		t_{30} = -0.84; P = 0.407
	***t*-test RT Neutral *vs* emotional**	**Subjects**
	t_{30} = -0.56; P = 0.577	All
	t_{20} = -0.21; P = 0.837	R Go>NoGo Neutral Pictures (N=21)
	z_{109} = 0.264; P = 0.791	R Go<NoGo Neutral Pictures (N=10)
	t_9 = -0.6161; P = 0.553	
	**RT Interaction Emotion *vs* Memory**	**Subjects**
	$F_{1,30}$ = 0.32; P = 0.859	All
	$F_{1,20}$ = 0.87; P = 0.362	R Go>NoGo Neutral Pictures (N=21)
	$F_{1,9}$ = 0.97; P = 0.351	R Go<NoGo Neutral Pictures (N=10)
**Exp 7 B**		t_{32} = -0.037; P = 0.971
	***t*-test RT Neutral *vs* emotional**	**Subjects**
	t_{32} = -1.51; P = 0.142	All
	t_{17} = -1.22; P = 0.237	R Go>NoGo Neutral Pictures (N=18)
	t_{14}= -1.02; P = 0.324	R Go<NoGo Neutral Pictures (N=15)
	**RT Interaction Emotion *vs* Memory**	**Subjects**
	$F_{1,32}$= 0.48; P = 0.494	All
	$F_{1,17}$ = 1.68; P = 0.213	R Go>NoGo Neutral Pictures (N=18)
	$F_{1,14}$ = 0.013; P = 0.910	R Go<NoGo Neutral Pictures (N=15)

Supplementary Table 2. Related to Supplementary Figure 2. Summary table of the statistics for the K responses in each experiment.

**Experiment 1**	
t-test recognition memory K responses Go *vs.* NoGo (hits-false alarms)	t_{30} = 0.55; P = 0.587
One-sample t-test Go	t_{30} = 0.69; P = 0.494
One-sample t-test NoGo	t_{30} = 0.24; P = 0.811
**Experiment 2**	
t-test recognition memory K responses Go *vs.* NoGo (hits-false alarms)	t_{37} = 1.12; P = 0.270
One-sample t-test Go	t_{37} = 1.64; P = 0.109
One-sample t-test NoGo	t_{37} = 2.93; P = 0.006
**Experiment 3**	
t-test recognition memory K responses Go *vs.* NoGo (hits-false alarms)	t_{25} = 0.70; P = 0.490
One-sample t-test Go	t_{25} = 0.70; P = 0.490
One-sample t-test NoGo	t_{25} = 0.01; P = 0.990
**Experiment 4**	
t-test recognition memory K responses Go *vs.* NoGo (hits-false alarms)	t_{21} = 0.76; P = 0.455
One-sample t-test Go	t_{21} = 0.76; P = 0.104
One-sample t-test NoGo	t_{21} = 0.76; P = 0.455
**Experiment 5**	
t-test recognition memory K responses Go *vs.* NoGo (hits-false alarms)	t_{20} = 2.09; P = 0.049
One-sample t-test Go	t_{20} = 1.09; P = 0.290
One-sample t-test NoGo	t_{20} = 1.27; P = 0.217
**Experiment 6**	
t-test recognition memory K responses Go *vs.* NoGo (hits-false alarms)	t_{27} = 0.63; P = 0.534
One-sample t-test Go	t_{27} = 1.05; P = 0.301
One-sample t-test NoGo	t_{27} = 0.41; P = 0.681
**Experiment 7 A**	
t-test recognition memory K responses Go *vs.* NoGo (hits-false alarms; collapsing across emotional and neutral stimuli)	t_{30} = 1.62; P = 0.115
One-sample t-test Go	t_{30} = 1.05; P = 0.301
One-sample t-test NoGo	t_{30} = -1.1487; P = 0.260
**Experiment 7 B**	
t-test recognition memory K responses Go *vs.* NoGo (hits-false alarms; collapsing across emotional and neutral stimuli)	t_{32} = -0.37; P = 0.715
One-sample t-test Go	t_{32} = 0.04; P = 0.970
One-sample t-test NoGo	t_{32} = 0.44; P = 0.660

Supplementary Table 3. Related to Figure 2. NoGo commission error rates and memory performance by number of preceding Go stimuli. Summary of the interaction term of repeated measures ANOVA for Exp 1-6. The linear contrast term for commission error rates by number of preceding Go stimuli is also given. Bayesian information criterion (BIC)-based estimation of posterior probabilities is provided to test evidence in favor the null hypothesis.

Exp	Commission errors by number of preceding Go stimuli	Linear Contrast	Memory performance for the NoGo stimuli by number of preceding Go stimuli
1	$F_{1.88,56.44}$ = 1.90; P = 0.162	$F_{1,30}$ = 5.44 ; P = 0.027; η^2 =0.349	$F_{2.34,70.16}$ =0.82; P =0.487<br>ΔBIC_{10} = 92.04 : $Pr_{BIC\ \sim 1}$
2	$F_{2.1,77.684}$ = 2.50; P = 0.086	$F_{1,37}$ = 4,64; P = 0.038; η^2 = 0.111	$F_{1.93,71.48}$ = 0.34; P =0.707<br>ΔBIC_{10} = 159.55 ; $Pr_{BIC\ \sim 1}$
3	$F_{1.685,42.13}$ = 0.87; P = 0.410	$F_{1,25}$ = 1.56; P = 0.220, η^2 = 0.059	$F_{2.09,52.17}$ =0.36 ; P =0.710<br>ΔBIC_{10} = 113.52 ; $Pr_{BIC\ \sim 1}$
4	$F_{1.7,35.75}$ = 2.96; P = 0.072	$F_{1,21}$ = 7.79; P = 0.011, η^2 = 0.271	$F_{2.09,43.97}$ =1.40 ; P = 0.260<br>ΔBIC_{10} = 59.30; $Pr_{BIC\ \sim 1}$
5	$F_{1.741,34,825}$= 2,34; P =0.118	$F_{1,20}$ = 3,93; P = 0.061, η^2 =0.164	$F_{2.53,20.66}$ = 0.06; P =0.968<br>ΔBIC_{10} = 103.75 ; $Pr_{BIC\ \sim 1}$
6	$F_{1.3,34.97}$ = 3.55; P = 0.058	$F_{1,27}$ = 4.72; P = 0.390, η^2 =0.149	$F_{1.75,47.38}$ = 1.42; P =0.250<br>ΔBIC_{10} = 143.35; $Pr_{BIC\ \sim 1}$

Supplementary Table 4. Go stimuli memory performance by number of preceding NoGo stimuli. One way ANOVA on the memory for the Go items depending on whether there are 0,1,2,3 or 4 preceding NoGo item for experiments from 1 to 6. Bayesian information criterion (BIC)-based estimation of posterior probabilities is provided to test evidence in favor the null hypothesis.

Exp	One Way ANOVA on memory performance	BIC-based estimation of posterior probabilities
1	$F_{1.98,\ 59.53}$ = 1.72 ; P = 0.188	ΔBIC_{10} = 92.04; $Pr_{BIC\ \sim 1}$
2	$F_{1.40,\ 52}$ = 0.61 ; P =0.49	ΔBIC_{10} = 109.94; $Pr_{BIC\ \sim 1}$
3	$F_{2.2,\ 55}$ = 0.36; P = 0.72	ΔBIC_{10} = 164.80; $Pr_{BIC\ \sim 1}$
4	$F_{2.05,\ 42.98}$ = 1.63 ; P = 0.21	ΔBIC_{10} = 96.39 ; $Pr_{BIC\ \sim 1}$
5	$F_{2.05,\ 41.07}$ = 0.16 ; P = 0.85	ΔBIC_{10} = 103.75 ; $Pr_{BIC\ \sim 1}$
6	$F_{1.85,\ 49.94}$ = 0.18 ; P = 0.82	ΔBIC_{10} = 143.35; $Pr_{BIC\ \sim 1}$

Supplementary Table 5. Related to Figure 3. Interaction Go/NoGo *vs.* subsequent memory fMRI effects. Data thresholded at P < 0.001 uncorrected. *SVC using Keren LC mask (SUIT space coordinates -2, -34, -23, Z = 3.32, P_{FWE} = 0.035). BA: Brodmann area

			MNI Coordinates			
**Structure**	**BA**	**Left/Right**	**X coord**	**Y coord**	**Z coord**	**Zscore**
Locus coeruleus*	-	R	2	-28	-16	3.38

Supplementary Table 6. Related to Figure S2. Go *vs.* NoGo fMRI effects. Data thresholded at P < 0.001 uncorrected. *FWE-corrected for the whole brain cluster level P < 0.05. Activations at peak level with more than 5 voxels cluster. BA: Brodmann area

			MNI Coordinates			
**Structure**	**BA**	**Left/Right**	**X coord**	**Y coord**	**Z coord**	**Zscore**
Caudate (tail)	-	L	-16	-24	18	4.31
Superior Parietal Lobule *	5	L	-30	-46	68	4.16
Anterior Lateral Intraparietal Sulcus *	40	R	56	-32	54	4.00
Premotor Cortex	6	L	-28	0	66	3.91
Postcentral Gyrus	7	R	4	-56	72	3.86
Caudate (body)	-	R	8	0	22	3.83
Cuneus	18	R	2	-98	18	3.72
Anterior Lateral Intraparietal Sulcus	2	L	-56	-30	52	3.70
Postcentral Gyrus	2	L	-66	-22	28	3.55
Cuneus	19	R	2	-94	28	3.55
Postcentral Gyrus	7	L	-14	-50	74	3.37

Supplementary Table 7. Related to Figure 3. Memory (R vs. F) fMRI effects. Data thresholded at *P* <0.001 uncorrected. † SVC using bilateral posterior parahippocampal mask converted to MNI space from Harvard-oxford atlas for fsl (-24 -28 -20, Z = 3.51, P_{FWE} =0.05). BA: Brodmann area

			MNI Coordinates			
**Structure**	**BA**	**Left/Right**	**X coord**	**Y coord**	**Z coord**	**Zscore**
Middle Temporal Gyrus	21	L	-56	-6	-18	4.63
Caudate (body)	-	R	12	-2	26	3.97
Cingulate Gyrus	24	L	-8	-4	28	3.93
Fusiform	37	L	-26	-42	-16	3.84
Caudate (body)	-	L	-10	16	16	3.82
Parahippocampal Gyrus †	35	L	-24	-26	-20	3.73
Orbitofrontal Gyrus	47	L	-44	22	-4	3.72
Precentral Gyrus	4	R	60	-10	26	3.26

Supplementary Table 8. Related to Figure 3. PPI of the interaction between LC activation and Go/NoGo *vs.* subsequent memory fMRI effects. Data thresholded at *P* < 0.001 uncorrected. † SVC using bilateral posterior parahippocampal mask converted to MNI space from Harvard-oxford atlas for fsl (-32 -38 -12), Z = 3.79, P_{FWE} = 0.02. BA: Brodmann area

			MNI Coordinates			
**Structure**	**BA**	**Left/Right**	**X coord**	**Y coord**	**Z coord**	**Zscore**
Fusiform	37	L	-44	-40	-14	4.05
Parahippocampal † gyrus	37	L	-32	-38	-12	3.79
