# Supplementary material for: Action boosts episodic memory encoding in humans via engagement of a noradrenergic system
Source: Nat Commun. 2019 Aug 6;10:3534. doi: 10.1038/s41467-019-11358-8 (PMC6684634; doi:10.1038/s41467-019-11358-8)
Supplement: Supplementary file 2 — Description of Supplementary Files [file 41467_2019_11358_MOESM2_ESM.pdf]

DESPCRIPTION OF SUPLEMETARY FILES

SUPPLEMENTARY DATA 1

Please note that given the extension of the NoGo vs. Go fMRI effects table, this has been provided as an independent supplementary file named Supplemetary Data 1 in xlsx format. Supplementary Dataset 1 is related to Supplementary Figure 2. It displays NoGo vs. Go fMRI effects. Data thresholded at $P < 0.001$ uncorrected. *FWE-corrected for the whole brain cluster level $P < 0.05$. Activations at peak level with more than 5 voxels cluster. BA: Brodmann area (Excel format).

SUPPLEMENTARY DATA 2

A “Participant Database” is provided as a supplementary data named Supplementary Data 2.xlsx. In this spreadsheet we provide the most relevant behavioral results per every subject that participate in each of the 8 experiments (Exp 1 to 7 and 7 Replication) we conducted for this paper. There is one sheet corresponding to each experiment.

For Exp 1-6 there are 9 columns named “Subjects”, “GoR”, “NoGoR”, “GoK”, “NoGoK”, “FAR”, “FAK”, “MeanRT GoR” and “MeanRT GoK”.

1. Subjetcs: indicating a subject identifier.
2. GoR: calculated as the percentage of correct number of old Go trials correctly pressed at encoding and indicated as remembered items at recognition.
3. GoK: calculated as the percentage of correct number of old Go trials correctly pressed at encoding and indicated as familiar items at recognition.
4. NoGoR: calculated as the percentage of correct number of old NoGo trials correctly non-pressed at encoding and indicated as remembered items at recognition.
5. NoGoK: calculated as the percentage of correct number of old NoGo trials correctly non-pressed at encoding and indicated as familiar items at recognition.
6. FAR: False alarms defined as new items presented at recognition phase identified as old.
7. FAK: False alarms defined as new items presented at recognition phase identified as familiar.

8. MeanRT GoR: average reaction time for GoR trials (see definition 2. above).
9. MeanRT GoK: average reaction time for GoK trials (see definition 3. above).

For experiments 7 and 7 Replication please note definitions from 2 to 7 have been split into “Neu” and “Emo” referring to neutral and emotional trials respectively.

In each data sheet a legend with reasons why certain subjects were excluded is provided according with the established exclusion criteria (see Methods). The corresponding rows for each excluded subject is font colored accordingly.
