# Supplementary material for: Action boosts episodic memory encoding in humans via engagement of a noradrenergic system
Source: Nat Commun. 2019 Aug 6;10:3534. doi: 10.1038/s41467-019-11358-8 (PMC6684634; doi:10.1038/s41467-019-11358-8)
Supplement: Supplementary file 6 — Reporting Summary [file 41467_2019_11358_MOESM6_ESM.pdf]

nature research

Corresponding author(s): Mar Yebra

Last updated by author(s): Jun 12, 2019

Reporting Summary

Nature Research wishes to improve the reproducibility of the work that we publish. This form provides structure for consistency and transparency in reporting. For further information on Nature Research policies, see Authors & Referees and the Editorial Policy Checklist.

Statistics

For all statistical analyses, confirm that the following items are present in the figure legend, table legend, main text, or Methods section.

n/a	Confirmed	
☐	☒	The exact sample size (n) for each experimental group/condition, given as a discrete number and unit of measurement
☐	☒	A statement on whether measurements were taken from distinct samples or whether the same sample was measured repeatedly
☐	☒	The statistical test(s) used AND whether they are one- or two-sided<br>*Only common tests should be described solely by name; describe more complex techniques in the Methods section.*
☒	☐	A description of all covariates tested
☐	☒	A description of any assumptions or corrections, such as tests of normality and adjustment for multiple comparisons
☐	☒	A full description of the statistical parameters including central tendency (e.g. means) or other basic estimates (e.g. regression coefficient) AND variation (e.g. standard deviation) or associated estimates of uncertainty (e.g. confidence intervals)
☐	☒	For null hypothesis testing, the test statistic (e.g. F, t, r) with confidence intervals, effect sizes, degrees of freedom and P value noted<br>*Give P values as exact values whenever suitable.*
☒	☐	For Bayesian analysis, information on the choice of priors and Markov chain Monte Carlo settings
☒	☐	For hierarchical and complex designs, identification of the appropriate level for tests and full reporting of outcomes
☐	☒	Estimates of effect sizes (e.g. Cohen's d, Pearson's r), indicating how they were calculated

Our web collection on statistics for biologists contains articles on many of the points above.

Software and code

Policy information about availability of computer code

Data collection	Behavioral data was collected
Data analysis	Functional imaging data were analyzed using statistical parametric mapping (SPM8; http://www.fil.ion.ucl.ac.uk/spm) and the the SUIT toolbox (http://www.diedrichsenlab.org/imaging/suit.htm) was employed in order to improve the spatial reliability of the observed LC response. Raw pupil data were analyzed using Fieldtrip toolbox (http://www.fieldtriptoolbox.org/) and a basis function approach using custom code was used based on methods previously published.

For manuscripts utilizing custom algorithms or software that are central to the research but not yet described in published literature, software must be made available to editors/reviewers. We strongly encourage code deposition in a community repository (e.g. GitHub). See the Nature Research guidelines for submitting code & software for further information.

Data

Policy information about availability of data

All manuscripts must include a data availability statement. This statement should provide the following information, where applicable:
- Accession codes, unique identifiers, or web links for publicly available datasets
- A list of figures that have associated raw data
- A description of any restrictions on data availability

Provide your data availability statement here.

Field-specific reporting

Please select the one below that is the best fit for your research. If you are not sure, read the appropriate sections before making your selection.

☒ Life sciences ☐ Behavioural & social sciences ☐ Ecological, evolutionary & environmental sciences

For a reference copy of the document with all sections, see nature.com/documents/nr-reporting-summary-flat.pdf

Life sciences study design

All studies must disclose on these points even when the disclosure is negative.

Sample size	We used Cohen´s d meassure to estimate effect size in each experiment, we also used partial eta-squared for repeated meusures ANOVAs as the ratio of variance accounted for by the effect and that effect plus its associated error variance. For each experiment more subjects were needed until Cohen´s d across experiments were comparable.
Data exclusions	For all experiments, exclusion criteria were applied on the basis of task performance at encoding and recognition. Participants performing at less than 90% correct button press for Go, and correct withheld responses for NoGo, trials were excluded from analyses. For the fMRI experiment the threshold was 85%. Furthermore, those participants with poor memory performance (defined as correct hit remembered rate minus remember false alarm rate less than 0%) were not further considered for analysis. In addition, participants making button-press responses for less than 90% of trials during recognition testing were excluded from analyses.
Replication	Over a series of 7 experiments, we consistently replicated our experimental finding. We observed better memory for stimuli co-occurring with action. We conducted an aditional experiment and successfully reporduced findngs for experiment 7.
Randomization	Exp 7 B was performed as a replication of Exp 7 A. In the former subjects were included in the study until reaching an effect size of interest (interaction between emotion and action for subjects that show AIME) of at least 25%. For Exp 7 B the same stop criteria for including subjects in the study was applied preserving a similar sample size as in Exp 7 A. Using a stop criterion based on effect size has its limitations. For this reason, we further validated the statistical robustness of our results by applying a boot-strap procedure of 1000 iterations to the memory data in Exp 7A and 7B, using the MATLAB Resampling statistical toolkit.
Blinding	This is not relevant to our study. We did not separate our sample for the different experiments in groups.

Reporting for specific materials, systems and methods

We require information from authors about some types of materials, experimental systems and methods used in many studies. Here, indicate whether each material, system or method listed is relevant to your study. If you are not sure if a list item applies to your research, read the appropriate section before selecting a response.

Materials & experimental systems

n/a	Involved in the study	
☒	☐	Antibodies
☒	☐	Eukaryotic cell lines
☒	☐	Palaeontology
☒	☐	Animals and other organisms
☐	☒	Human research participants
☒	☐	Clinical data

Methods

n/a	Involved in the study	
☒	☐	ChIP-seq
☒	☐	Flow cytometry
☐	☒	MRI-based neuroimaging

Human research participants

Policy information about studies involving human research participants

Population characteristics	A total of 296 human subjects (aged 18-35; 116 female) were recruited via advertisement to participate in our study, which comprised 8 experiments. No individual performed more than one experiment. Participants were right- or left-handed for the behavioral experiments and all right-handed for the fMRI experiment, had no history of neurological or psychiatric disease, and normal or corrected-to-normal visual acuity.
Recruitment	All participants were recruited via online or personal advertisement via Universidad Politecnica de Madrid.
Ethics oversight	All participants provided written informed consent prior to commencement of the study. The study was approved by the ethical committee of the Universidad Politecnica de Madrid.

Note that full information on the approval of the study protocol must also be provided in the manuscript.

Magnetic resonance imaging

Experimental design

Design type

Event-related

Design specifications

One encoding session per experimental subject of 190 trials (95 Go and 95 NoGo). Stimuli were presented for 250ms with a variable ISI from 2.3 to 3.3s

Behavioral performance measures

Behavioral performance was measured by recording the following variables. Encoding: response (key pressed at encoding phase Go or absence of key pressed NoGo) and latency (RT). Recognition: key pressed corresponding to Remember, Know, Forgotten responses), and RT.
Subjects were performing the task as expected if they showed more than 85% of correct Go or NoGo trials at encoding or responses at recognition, and acceptable memory performance (defined as correct hit remembered rate minus remember false alarm rate less than 0%).

Acquisition

Imaging type(s)

Functional, structural

Field strength

3 Tesla

Sequence & imaging parameters

Structural: MPRAGE T1-weighted anatomical images with 1mm3 resolution (repetition time (TR), 2300 ms; echo time (TE), 2.98 ms; flip angle, 9°).
Functional: Gradient-echo echo-planar T2*-weighted MRI image volumes with blood oxygenation level-dependent contrast were acquired, plus five additional volumes, acquired at the start of each session and subsequently discarded, to allow for T1 equilibration effects. Each whole-brain volume comprised 40 axial slices (2.2mm thick; distance factor 0.25; repetition time 2.43 s; echo time 30 ms; flip angle 90°; FOV 192 mm x 192 mm; matrix 64 x 64) sequentially acquired (ascending)

Area of acquisition

Whole brain

Diffusion MRI ☐ Used ☒ Not used

Preprocessing

Preprocessing software

Functional imaging data were analyzed using statistical parametric mapping (SPM8; http://www.fil.ion.ucl.ac.uk/spm)
Each subject’s fMRI time series was realigned, slice time corrected, normalized to MNI space and smoothed with an isotropic 3D Gaussian kernel of 6 mm full-width half-maximum.

Normalization

Normalisation was performed using spm8 approach using functional images after realignment and slice time correction. Step 1: Linear affine transformation using a 12-parameter affine transformation performed automatically by minimizing squared distance between parameters and expected values. Step 2: Non-linear wraping using warps which are modelled by linear combinations of non-linear smooth discrete cosine transform basis functions.
For the second analysis, the SUIT toolbox uses nonlinear deformation.

Normalization template

Each subject’s fMRI time series was normalized to MNI space using the EPI.nii image provided by SPM as a template.
In a second analysis, the spatially unbiased atlas template of the cerebellum and brainstem (SUIT) atlas template was used.

Noise and artifact removal

Response errors were modeled separately. Six movement parameters were modelled as nuisance covariates

Volume censoring

n/a

Statistical modeling & inference

Model type and settings

Mass-univariate (GLM)
First level: Session-specific parameter estimates of the magnitude of the haemodynamic response for each stimulus type were calculated for each voxel in the brain. A contrast of parameter estimates modeling each

	comparison of interest (e.g., remembered vs. forgotten Go vs. NoGo images) was calculated in a voxel-wise manner to produce, for each subject, one contrast image for that particular effect.<br>Second level: For the random effects analysis, each subject's contrast image was entered into a one-sample t-test across participants. We report group-level analyses pertaining to the main effects and interaction term of our response (Go, NoGo) by subsequent memory (Remembered, Forgotten) 2 by 2 factorial design.
Effect(s) tested	To test for effects of motor action on memory, we specified 6 effects of interest in a general linear model (GLM): the events corresponding to Go and NoGo trials, separated according to whether these images yielded a subsequent remember (R), familiar with (K) or forgotten (F) response at recognition testing. Event-specific responses were modeled by convolving a delta-function with a canonical haemodynamic response function (HRF) to create regressors of interest. A factorial design was used.

Specify type of analysis: ☒ Whole brain ☐ ROI-based ☐ Both

Statistic type for inference<br>(See Eklund et al. 2016)	Voxel-wise
Correction	Some of the results reported were non corrected, others were FWE corrected for whole brain or small volume corrected (for the latter, using probabilistic atlases)

Models & analysis

n/a	Involved in the study
☐	☒ Functional and/or effective connectivity
☒	☐ Graph analysis
☒	☐ Multivariate modeling or predictive analysis

Functional and/or effective connectivity	A psychophysiological interaction (PPI) analysis to estimate contextspecific changes in correlation between the LC and the rest of the brain. Specifically, we tested which regions were functionally connected with LC under the experimental context of successful encoding between Go vs. NoGo trials.
